# Pregnancy-Associated Glycoproteins Identification in Skopelos Goat Milk by Means of Mass Spectrometry

**DOI:** 10.3390/vetsci12111092

**Published:** 2025-11-17

**Authors:** Efterpi Bouroutzika, Ekaterini K. Theodosiadou, Stavros Proikakis, Irene Valasi, George Th. Tsangaris

**Affiliations:** 1 Laboratory of Physiology, Faculty of Veterinary Science, University of Thessaly, Trikalon 224, 43100 Karditsa, Greece; evalasi@uth.gr; 2 Laboratory of Food Quality Control & Hygiene, Department of Food Science & Technology, Agricultural University of Athens, Iera Odos 75, 11855 Athens, Greece; stavrospro@aua.gr; 3 Proteomics Research Unit, Biomedical Research Foundation of the Academy of Athens, 11527 Athens, Greece; gthtsangaris@bioacademy.gr

**Keywords:** pregnancy-associated glycoproteins, goat, caprine milk, mass spectrometry, proteomics

## Abstract

The proteins most frequently associated with pregnancy are the pregnancy-associated glycoproteins (PAGs), which are abundantly expressed placental products in species belonging to the order Cetartiodactyla. Multiple PAG isoforms are expressed across different species and stages of gestation, with their expression influenced by factors, such as breed and bodyweight. These proteins can be detected in both blood and milk samples using ELISA or RIA assays, serving as early indicators of pregnancy. The present study aimed to detect PAGs in caprine milk through a non-invasive, high-throughput, mass-spectrometry-based proteomic approach. Milk samples were collected from 20 Skopelos breed goats at 20 and 45 days post-mating. Following appropriate processing for whey protein extraction, the samples underwent tryptic digestion to generate peptides for LC-MS/MS analysis. The proteomic investigation identified five distinct PAGs (caPAG_2_ on day 20 and caPAG_3_, caPAG_5_, caPAG_6_, and caPAG_12_,on day 45 post-mating) annotated to *Capra hircus*, along with 22 additional proteins associated with the fetal–maternal interface, pregnancy progression, and immune-related pathways. These findings demonstrate that LC-MS/MS provides a non-invasive, sensitive, and reliable method for detecting PAGs in caprine milk during the early stages of pregnancy (day 20, as the presence of caPAG_2_ revealed), applicable to both individual animals and flock-level monitoring.

## Simple Summary

This study is an attempt to identify pregnancy-associated glycoproteins (PAGs) by means of proteomics methods, specifically LC/MS-MS, in caprine milk. PAGs are specific proteins expressed in the placenta of Cetartiodactyla-order species, including all ruminants, during pregnancy. Milk samples from 20 Skopelos breed goats were analyzed. Five different caprine PAGs (caPAGs) were identified on days 20 and 45 as well as twenty-two other pregnancy-related proteins at the studied time points. The results show LC/MS-MS is a reliable and non-invasive tool for accurate early pregnancy diagnosis in goats via detection of PAGs in caprine milk samples.

## 1. Introduction

Caprine milk recently is used as an infants’ formula substitute in children with allergy to cow milk [1]. The higher content of caprine milk in essential fatty acids makes it easily digested compared to bovine milk. In addition, it contains proteins, such as lactadherin, with antimicrobial properties and roles in the prevention and treatment of intestinal injury in infants [2,3]. Moreover, caprine milk is used for the production of many Protected Designation of Origin cheeses, such as feta, or highly consumed, such as chèvre (goat cheese). For those reasons, the early determination of pregnancy status in goats as soon after mating as possible is necessary [4,5,6] using various indicators, such as pregnancy-associated glycoproteins (PAGs).

Pregnancy-associated glycoproteins (PAGs) are abundantly expressed placental products in species belonging to the Cetartiodactyla order [7,8,9,10,11], and they were firstly reported and studied in cattle approximately 30 years ago. They originate from the binucleated giant trophoblasts, which come from mononucleated trophoblasts, via mitotic polyploidy, and appear around the end of third week of gestation [12,13,14]. After their formation, each mature binucleated cell migrates across the microvillar junction and fuses with a uterine epithelial cell, forming a syncytial fetalmaternal cell that contributes to the development of multinucleated syncytia. In small ruminants, such as sheep and goats, the syncytia continue throughout pregnancy [15,16]. The presence of binucleated trophoblasts, along with their characteristic fusogenic properties, distinguishes the ruminant placenta from the epitheliochorial type. As a result, ruminants exhibit a unique capacity to secrete placental proteins directly into the maternal circulation. Numerous proteins, including PAGs, placental lactogen hormone, and prolactin-related proteins, are stored within the secretory granules of binucleated trophoblasts [10,17,18,19,20]. Upon release of these granules, the proteins expressed by binucleated cells are transferred into the maternal uterine connective tissue and subsequently enter the maternal bloodstream [21,22,23,24]. Certain PAGs are expressed in all trophoblasts (both mononucleated and binucleated), whereas others are restricted exclusively to the binucleated trophoblast giant cell population [24].

PAGs are pepsin-like aspartic proteinases expressed by mononuclear trophoblasts in several even-toed ungulate species and by binuclear giant cells in cattle and sheep [24,25,26]. They were first identified as pregnancy-specific blood antigens in cattle and were designated as pregnancy-specific protein B (PSPB) [21]. Their elevated concentration in blood plasma during early gestation facilitated the development of pregnancy detection assays, which are now commercially available for various domestic and wild ruminant species. It was estimated that there are up to 100 PAG genes and pseudogenes with more than 20 distinct transcripts identified to date [24]. However, the role of PAGs as active proteases remains unclear. PAGs are expressed starting around maternal recognition of pregnancy, and their expression rises throughout gestation, with blood concentrations peaking near parturition. High blood concentrations persist for several weeks to months after parturition due to the long half-life of PAGs in the blood. Many studies have implied the role of PAGs in immune function alterations during pregnancy, but this has not been clarified yet [24,26]. The extensive gene duplication observed among modern PAGs in ruminants, coupled with the apparent conservation of a functional peptide binding cleft, provides compelling evidence that these proteins may possess an as-yet unidentified role in ruminant pregnancy. Given their abundant expression at the fetal–maternal interface and their persistently high concentration in maternal blood plasma throughout gestation, further research is required to determine whether PAGs are indispensable molecules for the maintenance of ruminant pregnancy [27].

PAGs are the best index for early pregnancy diagnosis. This fact set the basis for the development of pregnancy ELISA and RIA kits, which are commercially available for several domestic and wild ruminants. Apart from blood circulation, PAGs cross the mammary barrier and can be detected in caprine milk [28]. However, the methods for the identification of PAGs in caprine milk through classic laboratory assays, such as ELISA or RIA, are not yet sensitive or precise. Thus, this study aimed to establish a proteomic method, an LC-MS/MS technique, as a reliable tool for early pregnancy diagnosis via detection of PAGs in caprine milk.

## 2. Materials and Methods

### 2.1. Experimental Overview

In total, 20 Skopelos breed goats, 2–4 years old, from one flock (EL-41BIO/exp-06), located in central Greece, participated in this study. Reproductive control was applied to all goats following a standard protocol with intravaginal insertion of progestogen sponges [60 mg medroxyprogesterone acetate (MAP, Ovigest, Hipra, Spain)] for 17 days, intramuscular administration of equine chorionic gonadotropin [350 IU eCG (Intergonan, Intervet, The Netherlands) at sponges’ removal, natural mating using 3 Skopelos bucks of known and proven fertility after estrus detection by teaser bucks every 6 h. The teaser bucks were inserted again in the goats in order to detect another estrus cycle in case some of them experienced early fetal losses and estrous cyclicity again, but none of these happened. The beginning of mating was set as day 0 (D0). At day 20 (D20) and day 45 (D45), morning milk samples were collected by hand in a 50 mL plastic volumetric container at the milking house, keeping the same order in goats and in hours, according to the technique described by Bouroutzika et al. [29]. The milk samples from each goat were initially separated in aliquots of 1 mL and then were stored at −20 °C until assayed. Pregnancy diagnosis was performed using transabdominal ultrasonography (SonoVet 2000; 4.5 to 6 MHz convex transducer; Medison CO, Seoul, Republic of Korea) at day 36 (D36) and at day 45 (D45).

This study was conducted according to the conditions prescribed by the European Council Directive 2010/63/EU for animal experimentation procedures and approved by Veterinary Directorate of Thessaly, Larissa, Greece (license number: 351974/12-09-2022).

### 2.2. Sample Preparation

The milk samples from the 20 goats arrived at the laboratory in a frozen state (−20 °C) for LC/MS-MS analysis. After thawing, 1 mL of the sample was centrifuged at 4000× *g* at 4 °C for 60 min. The resulting fat layer was removed, and the samples were pooled according to sampling time after successful pregnancy diagnosis via ultrasonography on D45. The skim milk was transferred to sterile 1.5 mL microcentrifuge tubes, and centrifugation was performed at 5500× *g* for 30 min at 4 °C for final separation in three layers (lipid, whey, casein layer). Then, the whey segment was extracted, and the protein content was assayed using the Bradford method [30]. According to Bradford assay, the milk samples were diluted at 1/20, and then 990 μL of Bradford reagent was added in an aliquot with 10 μL of the sample. Then the mixture was lightly shaken and remained in the dark for 3 min, and its absorbance was measured at 595 nm with a spectrophotometer [31]. The protein concentration of every sample was calculated according to the standard curve, and it was set at 250 μg of protein.

Protein extraction and peptide generation were performed using the same method in the main steps, as described by Velentzas et al. [32]. In brief, 200 μg of whey fraction were treated with 7 M urea buffer and 80 mM triethylammoniumbicarbonate (TEAB) under mild sonication in a water -bath for 30 min. The steps for protein reduction and alkylation were performed using dithiothreitol and iodoacetamide solutions (10 mM and 55 mM, respectively). Finally, a tryptic digestion of extracted proteins was necessary in order to generate peptides. Trypsin (Roche Diagnostics, Basel, Switzerland), at a final concentration of 500 ng/μL, was added to all samples in a humidified atmosphere, followed by an overnight digestion. The procedures were performed in triplicate as was the analysis of samples. All chemicals used were purchased from Sigma Chemical Co. (Burlington, MA, USA), unless otherwise stated.

### 2.3. LC-MS/MS Analysis

An LTQOrbitrap Elite coupled to a Dionex 3000 HPLC system (Thermo Scientific, Rockford, IL, USA) was used to perform the analysis of the digested samples. LC separation of peptides was performed on two Thermo Scientific columns (PepMap^®^ RSLC, C18, 100 Å, 3 μm bead-packed 15 cm column and 2 μm bead-packed 50 cm column) at a flow rate of 3 nL/min. The mobile phases A and B were 0.1% formic acid in water and 99% acetonitrile in water, respectively. The gradient elution profile was as follows: 2.0% B (98.0% A) for 10 min, 2.0–35.0% B (98.0–65.0% A) for 325 min, 80.0% B (20.0% A) for 10 min, 2.0% B (98.0% A) for 10 min. Data were collected in data-dependent MS/MS mode, using a standard top 10 method. Full scanning occurred at a resolving power of 60,000 with a maximum integration time of 250 ms. Scan range was fixed at 250 to 1250 *m*/*z*, and peptide fragmentation was performed in higher-energy collision dissociation (HCD) mode with a normalized collision energy of 36%. MS/MS spectra were acquired with 15,000 resolving power and a maximum integration time of 120 ms. Measurements were performed using *m*/*z* 445.120025 as lock mass. Dynamic exclusion settings were set to repeat count 1, repeat duration 30 sec, exclusion duration 120 sec, and exclusion mass widths of 0.6 *m*/*z* (low) and 1.6 *m*/*z* (high). Proteome Discoverer software (Thermo Scientific, Rockford, IL, USA) was the tool used for the *.raw data analysis using the *Capra hircus* and *Ruminantiae* for caprine milk *.fasta databases applied with the Sequest search engine. MS/MS searches were performed using a 20 ppm parent ion mass tolerance and a 0.05 fragment mass tolerance. Trypsin was the selected cleavage enzyme, with up to 2 missed cleavage points. Cysteine methylthio modification was selected as a fixed modification and oxidation of methionine as a variable. Peptide identifications were considered valid at 1% false discovery rate (q-value b0.01; percolator maximum Delta Cn was 0.05). The minimum length of acceptable identified peptides was set as 6 amino acids [30].

### 2.4. Bioinformatic Analysis

All identified milk proteins were annotated with their corresponding gene symbols using the UniProt Knowledgebase (http://www.uniprot.org, accessed on 6 November 2025–last visit). Functional classification of these proteins was conducted according to their Gene Ontology (GO) annotations, encompassing molecular function, biological process, and subcellular localization. Analyses included all detected milk proteins; in cases where multiple annotations were available, all relevant functional assignments were incorporated into the results [30]. Proteins were classified using the GO functional annotations for biological process, molecular function, and subcellular component. Functional relationship analysis of the differentially expressed proteins was performed using the STRING v.10 database (Search Tool for the Retrieval of Interacting Genes/Proteins, http://string-db.org, accessed on 6 November 2025–last visit).

## 3. Results

### 3.1. Pregnancy-Associated Glycoproteins in Caprine Milk on D20 and D45

The ultrasonographic (U/S) examination for pregnancy diagnosis that was performed at D36 after mating in goats revealed that all animals (20/20) were pregnant and at the same stage of pregnancy. The U/S examination 10 days later also confirmed that pregnancy in all goats was progressing physiologically.

The LC/MS-MS analysis revealed five PAGs originated from *Capra hircus*. caPAG_2_ was found on D20 and caPAG_3_, caPAG_5_, caPAG_6_, and caPAG_12_ on D45. The list with PAGs and the peptides that led to their identification are presented at Table 1.

### 3.2. Proteins Related to Early Pregnancy in Caprine Milk on D20 and/or D45

The LC/MS-MS analysis revealed the presence of several proteins related to various stages of early pregnancy, embryo implantation in the uterus, and placenta formation at the two studied time-points, D20 and/or D45. The most characteristic proteins related to early pregnancy and their peptides are listed in Table 2 and in more detail in the Supplementary Material. Briefly, 12 proteins were obtained on D20, 6 on D45, and 4 on both D20 and D45.

### 3.3. Bioinformatic Analysis

**Figure 1:**
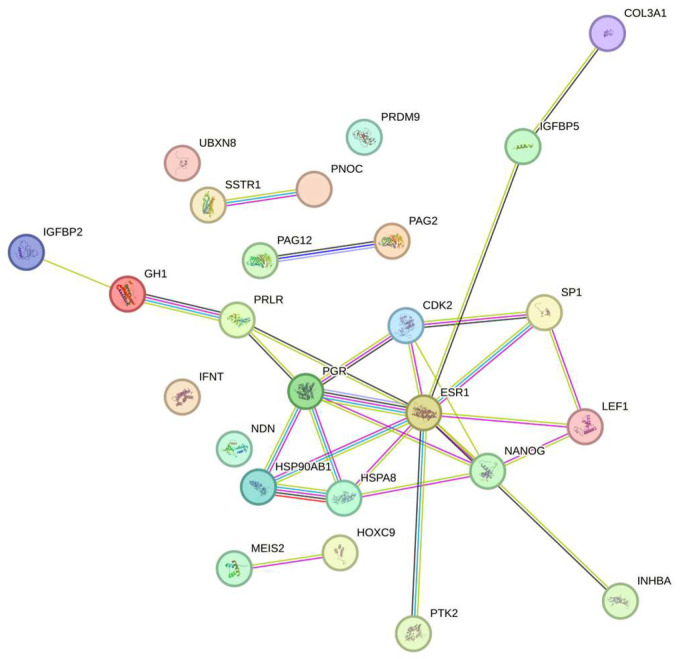
The network of proteins presented in Table 1 and Table 2.

The STRING analysis (Figure 1) revealed the interactions among the proteins, which are presented in Table 1 and Table 2.

## 4. Discussion

In this study, the PAG family in caprine milk was detected for the first time using mass spectrometry methods. From the five caPAGs detected in caprine milk, caPAG_2_ was detected during early pregnancy (D20), while caPAG_3_, caPAG_5_, caPAG_6_, and caPAG_12_ were detected on D45 post-mating. This innovative approach emphasizes the potential of advanced analytical methods for detecting and characterizing PAGs with precision in milk, a biological fluid that can be effortlessly obtained through non-invasive procedures. Goats enter estrus every 21 days, so the detection of pregnancy at day 21 would be valuable management tool. Usually, the pregnancy in goats is diagnosed by real-time B-mode ultrasonography via transabdominal or trans-rectal imaging techniques not before day 22 of gestation [33,34]. However, in goats, pathological conditions, such as hydrometra with small accumulations of fluid in the uterus, might be misdiagnosed as early pregnancy since placentomes or fetuses are not clearly distinct before day 30 of gestation [35]. Another pathological condition that can be falsely recorded as pregnancy in goats is early fetal loss, because it cannot be detected not even with trans-rectal ultrasonographic examination, since the fetal heartbeat can be recorded after day 35, leading to false positive pregnancy diagnosis [35,36]. This obstacle regarding successful pregnancy diagnosis without false positive results may be overcome with PAG identification in milk via LC/MS-MS, as the results of the present study indicated in already-pregnant goats.

Various hormonal assays have been tested using milk samples for early pregnancy diagnosis in goats, considering progesterone, estrone sulfate, and placental lactogen hormone assays. Progesterone assays of milk at days 20 to 22 post-breeding is not pregnancy-specific and may lead to false positive diagnoses, since low progesterone levels at this period indicate a non-pregnant status, while stable or rising levels are indicators of pregnancy [4]. Estrone sulfate or placental lactogen hormone assays cannot be applied for early pregnancy diagnosis, even though the detected hormones are pregnancy-specific [4].

PAGs are the best hormones for diagnosing pregnancy because they represent the specific indicators of pregnancy and feto-placental health status in ruminants. Using heterologous RIA, ovPAGs and ovPSPB can be detected in the blood of pregnant ewes as early as day 20 post-mating [37,38]. Throughout gestation ovPAG concentrations fluctuate depending on factors such as the ewe’s breed as well as the sex and number of developing fetuses [37,39]. A similar heterologous RIA in blood has been used in goats, with boPAG-1 and PSPB as standards and antisera raised against the ovine antigens. In this case, pregnancy could be detected by day 24 or 25 [4]. In the same study [4], other RIA techniques detecting caPAG_55+59_ and caPAG_55+62_ in blood were used for pregnancy diagnosis on day 21 or 22 with high accuracy. However, in caprine blood (serum or plasma) or milk samples, neither specific ELISA nor RIA methods have been applied for PAG identification, according to the available literature databases. The existing commercially available kits for these techniques, that are reasonably priced, are usually based on bovine PAGs; no kits are available for the precise and species-specific detection of caprine PAGs using blood or milk samples. Finally, ELISA kits for all ruminants’ PAGs in blood or milk, PAGs in goats can be accurately identified 28 days post-breeding.

PAGs are placental antigens and consist of several heavily glycosylated and isoelectric variants, each with a molecular mass of about 67 kDa. The presence of PAGs in blood serum is a useful indicator of pregnancy as the antigens enter the maternal bloodstream at around the time of implantation. PAG concentration rise steadily as gestation proceeds and peaks just before parturition, when it can reach 1 μg/mL or greater [9,22]. A group of nine PAGs (caPAG_1_, caPAG_3-7_, caPAG_9-11_) were expressed after day 45 of pregnancy and were localized to trophoblast binucleated cells. On the other hand, caPAG_2_ was detectable in blood plasma only in early pregnancy (after day 16) and expressed throughout trophectoderm [9,40]. The latter studies are in accordance with our findings, as caPAG_2_ was detected on D20 after mating, whereas the rest of them were found on D45.

The proteomics analysis of caprine milk revealed the presence of several other proteins that are involved in the establishment of pregnancy. The Interferon-tau (IFN-tau) found on D20 is responsible for maternal recognition of pregnancy, which happens in goats on day 18 after fertilization [41]. IFN-tau is secreted from day 15 after fertilization until day 24 of pregnancy, meaning that the cytokine is early-pregnancy-specific only in ruminants with a short time period for detection [42]. IFN-tau suppresses the release of prostaglandin F_2α_ and is involved in prostaglandin synthesis, resulting in increased levels of progesterone and hindering the regression of the corpus luteum, both crucial elements for the continuance of pregnancy in goats [41]. Lymphocyte enhancer-binding factor-1 (Lef-1) is also involved in the chorio-allantoic fusion necessary for embryo implantation in the uterus via trophectoderm; the morphogenesis of radial glial scaffolds, paraxial mesoderm and limbs in embryos; and in the development of mammary and endometrial glands [43,44]. In trophoblast fusion and placenta formation, a key role is played by the Envelope protein syncytin-rum1, which exerts fusogenicity in maternal cells in order to form the placentomes [45]. Another protein of interest found on D45, supporting the fact that the goats were pregnant at the studied time points, is PFOXic. This protein is expressed in developing goat ovaries from day 36 post-mating until they reach adulthood [46]. Moreover, Heat Shock Protein 90 (HSP90) is known to be a chaperone that stabilizes the inactive forms of steroid hormones receptors until their binding to the hormones is completed [47]. The expression of HSP90 is found to take place during the whole pregnancy and especially in intermediate trophoblast, syncytiotrophoblast and cytotrophoblast cells [47]. The presence of NANOG (Homeobox protein) transcription factor on D45 reveals the existence of pluripotent embryonic stem cells, and it is also exclusively detected in epiblasts during the implantation of blastocysts [48]. Lastly, INHB (Inhibin beta A chain) is known to be involved in endodermal cell differentiation, mesodermal cell differentiation, roof of mouth development, and striatal medium spiny neuron differentiation and in pathways related to progesterone and follicle-stimulating hormone secretion as well as female and male gonad differentiation and development [49,50].

Mass-spectrometry-based proteomics depends on effective protein fractionation followed by ionization and MS-based fragmentation of proteins or peptides [51]. The resulting peptide fragmentation pattern reveals its amino acid sequence and potential post-translational modifications. Through comparison with reference databases, particularly SWISS-PROT and PFAM, these peptides can be accurately matched to their corresponding full-length proteins [52,53]. The milk proteome and peptidome of the Skopelos breed of *Capra hircus* was identified partially (see Supplementary Material) using metaproteomics and metapeptidomics analysis and; keeping in mind the complexity of pregnancy they led to the identification of peptides derived from PAGs. Their discovery in milk can lead to early pregnancy diagnosis via LC/MS-MS. Also, the detection of proteins related to pregnancy, such as prolactin and progesterone receptors, support the accuracy of pregnancy diagnosis. However, the database regarding the proteomes of *Capra hircus* and especially the different breeds of goats, including Skopelos, is not fully complete for blood and milk samples making the use of other species databases absolutely necessary, such as those for *Ovis aries* and *Bos Taurus*, both ruminants in the Cetartiodactyla order. This limitation will be addressed when the proteome mapping of these species is completed, and the use of core peptides leading to species-specific protein identification will be widely accepted.

Additionally, the proteome represents the current health status of the goat, which can be associated with pregnancy too. Ott [27] tried to elucidate the role of immune modulation during the early stages of gestation, and the findings are in agreement with the identified immune-related proteins derived from caprine milk in the present study. Indeed, the majority of the aforementioned proteins in Table 2, in addition to their direct link to progression of pregnancy, demonstrate immune-related properties. Prolactin receptor is involved in cytokine-mediating signaling pathway and humoral immunity, since the receptor is expressed in a great variety of immune cells, including macrophages, monocytes, lymphocytes, granulocytes, natural killer cells, and thymic epithelial cells [54]. In addition, Lef-1 is part of cytokine-mediating signaling pathway and controls interleukin-4 expression and proliferation of B cells [55,56,57]. Moreover, Akirin 2 is known to be involved in formation of cerebral cortex as it is expressed in embryonic telencephalon [58] and concomitantly regulates inflammatory gene transcription in innate cells via bridging the NF-κB and SWI/SNF complexes [59]. The fact that Akirin 2 controls the SWI/SNF complex shows the important role of the protein in regulation and proliferation of B cells [59]. Lastly, Growth Hormone receptor participates in cytokine signaling pathway and relates to interleukin-1 [60]. These findings reinforce the assumption that caprine milk is a product rich in elements with immune properties and parts of immune-related pathways. The latter is the reason that consumption of caprine milk is growing, as is that of the dairy products produced from it.

This work has some limitations that must be addressed in future research. The concentration of PAGs during the early days of pregnancy (caPAG_2_ on D20) and their variation in the same period both in blood and milk samples. An attempt to compare the LC/MS-MS technique with the standard ELISA and/or RIA methods regarding PAG identification in blood and/or milk of goats as pregnancy diagnostic tools is already underway in order to minimize all concerns regarding the precision of the proteomic method. Also, the method will be standardized in caprine milk regardless of breed, so it can be widely used as a low-cost, precise, sensitive, and effective diagnostic tool for advanced management in highly productive flocks.

## 5. Conclusions

This study indicated, for the first time, the use of LC-MS/MS as a non-invasive and sensitive method for the detection of PAGs in caprine milk, even during early stages of pregnancy, in individual goat(s), a group, or a flock. From the five caPAGs detected in caprine milk, caPAG_2_ was detected during early pregnancy (D20), while caPAG_3_, caPAG_5_, caPAG_6_, and caPAG_12_ were detected on D45 post-mating. Furthermore, proteins related to early pregnancy can be identified, demonstrating immunomodulatory properties and underscoring the connection between normal pregnancy progression and the immune system. This sensitive, precise, and low-cost approach can be utilized in goat flocks for early pregnancy diagnosis and for monitoring its progression safely, since the late detection of early loses has a significantly negative economic effect both on the farmer and goat dairy product price. This research paves the way for further investigation into the structural and functional roles of PAGs and other pregnancy-related proteins, contributing to a deeper understanding of their inter-relationship and biological significance.

## Figures and Tables

**Table 1:** Pregnancy-associated glycoproteins (PAGs) and their peptides found on day 20 (D20) and on day 45 (D45) of pregnancy in milk samples of Skopelos goats.

No.	PAGs	Peptides	Days
1	caPAG_2_	LNWIPVSQTKSWLITVDR	D20
2	caPAG_3_	AGDWSVR	D45
		DSNVTIVPLRNMR	
3	caPAG_5_	DKQEGSVVmFGGVDHR	
4	caPAG_6_	IKGKVVHDTVR	
5	caPAG_12_	KTLSGKHMLNNFLK	

**Table 2:** Proteins related to early pregnancy and their peptides found on day 20 (D20) and/or day 45 (D45) in milk samples of Skopelos goats.

D20	
Protein Name	Peptides
Envelope protein syncytin-rum1	KAVLQNRMALDILTAAQGGTcAIIK
Truncated estrogen receptor alpha	LASTSDKGSMAMESAK
Progesterone receptor	GEAAEGAAVRPPEK
Cyclin-dependent kinase 2	TLGTPDEVVWPGVTSmPDYKPSFPK
Interferon-alpha	AEVmRAFSSSTNLQERFR
Collagen type III alpha 1	DGTSGHPGPIGPPGPR
Prepronociceptin	VmARGSWQLSPADPDHVAAAPDQAR
Somatostatin receptor 1	TAANSDGTVAcNMLMPEPAQR
Interferon tau BB12	TEPGLEEVGDMEQK
	DFAFPQEMVEGGQLQEAQAISVLHEmLQQSFNVFHPER
SP1 transcription factor	IEKGVGGNNGGNGNGSGAFSQAR
Lymphocyte enhancer-binding factor-1 isoform 2	EKLQESASGTGPR
Heat shock cognate 71 kDa protein	SINPDEAVAYGAAVQAAILSGDK
	SQIHDIVLVGGSTR
	TTPSYVAFTDTER
	NQVAMNPTNTVFDAK
	QTQTFTTYSDNQPGVLIQVYEGER
	MVNHFIAEFK
	IINEPTAAAIAYGLDK
	FEELNADLFR
**D20 and D45**	
Prolactin receptor	VTDSNILVLIPDPQAQKKK
NANOG (homeobox protein)	mSATGPISNYYVDSLISHDNEDLLASR
Growth hormone receptor	MLILPPVPVPK
Heat shock protein HSP 90-beta	YHTSQSGDEMTSLSEYVSR
	YHTSQSGDEmTSLSEYVSR
	NPDDITQEEYGEFYK
	HFSVEGQLEFR
	GVVDSEDLPLNISR
	SLTNDWEDHLAVK
	IDIIPNPQER
	ELISNASDALDK
**D45**	
PFOXic protein	QGSPKLTSLNTILHPPLK
Insulin-like growth factor binding protein 5	IAERDSREHEEPTTSEMAEETYSPK
Meis homeobox 2	QRVQNVHGINGSSISSAESR
Necdin	EITKmQIMEFLARVFK
PRDM9	THTGEKPYVcREcGRcFSDK
INHB (Inhibin beta A chain)	RAEmNELmEQTSEIITFAESGTAR

## Data Availability

The original contributions presented in this study are included in the supplementary material. Further inquiries can be directed to the corresponding authors.

## Supplementary Materials

The supporting information can be downloaded at https://www.mdpi.com/article/10.3390/vetsci12111092/s1.
